# Time-Series Transcriptome Reveals Differential Regulation of Flavonoid Biosynthesis in *Dendrobium huoshanense* Under Musical–Acoustic Stimuli: Insights for Green Horticultural Production

**DOI:** 10.3390/genes17101178

**Published:** 2026-09-24

**Authors:** Mei Huang, Yinhua Liu, Mengru Liu, Yuxiao Ling, Yeyu Yin, Tingting Wang, Wei Wang, Shanyong Yi

**Affiliations:** Department of Arts, Traditional Chinese Medicine Institute of Anhui Dabie Mountain, West Anhui University, Lu’an 237061, China; hmcd--1999@163.com (M.H.); 19045501287@163.com (Y.L.); lmr010806@163.com (M.L.); lyx13505694168@163.com (Y.L.); b612868@163.com (Y.Y.); 13886609204@163.com (T.W.)

**Keywords:** *Dendrobium huoshanense*, transcriptome, flavonoid biosynthesis, abiotic stress, gene expression

## Abstract

Background: Sound vibration is an emerging physical signal that influences plant growth and secondary metabolism; however, the genome-wide transcriptional responses of valuable medicinal orchids to complex acoustic stimuli remain largely uncharacterized. Methods: Here, we performed time-series RNA-seq on acclimatized plantlets of *Dendrobium huoshanense* exposed to two musically structured acoustic stimuli with contrasting acoustic characteristics (*Forest Fantasy* and *Erquan Yingyue*) plus a no-music control; leaf samples were collected at 1, 6, 12, and 24 h, and quantitative real-time polymerase chain reaction (qRT-PCR) was used to validate the transcriptome data. Results: The two acoustic treatments triggered distinct transcriptional reprogramming of flavonoid biosynthesis. Early biosynthetic genes *chalcone synthase* (*CHS*) and *naringenin 7-O-methyltransferase* (*NOMT*) were rapidly up-regulated under *Forest Fantasy*, whereas late-step modification genes (*naringenin 3-dioxygenase* (*F3H*), *flavonoid 3′-monooxygenase* (*F3′H*), *hydroxycinnamoyl-CoA shikimate/quinate hydroxycinnamoyl transferase* (*HCT*), *caffeoyl-CoA O-methyltransferase* (*CCoAOMT*)) were persistently suppressed by *Erquan Yingyue*. Differentially expressed gene (DEG)-derived hub structural genes (*cinnamate 4-hydroxylase* (*C4H*), *F3′H*, *CCoAOMT*) and co-expressed transcription factor candidates (MYB, WRKY, NAC) were identified via Pearson correlation-based network analysis. Conclusions: These results indicate that *D. huoshanense* can differentiate complex musical signals at the transcript level, suggesting short-term natural-style acoustic stimulation as a promising potential horticultural strategy for quality improvement of this precious medicinal orchid.

## 1. Introduction

*D*. *huoshanense* is a rare and endemic medicinal orchid in China. Its stems are rich in polysaccharides, flavonoids, alkaloids, phenols, phenanthrenes, and bibenzyl compounds [1], which exert multiple pharmacological effects including antioxidant activity, immunomodulation, and hepatoprotection [2,3,4]. A series of flavonoid derivatives has been isolated and identified from *D. huoshanense*, such as naringenin, rutin, isoschaftoside, schaftoside, vitexin-2″-*O*-*β*-D-glucopyranoside, and apigenin-6,8-di-*C*-glycosides [5]; the total flavonoid content in stem tissues varies from 2 mg/g to 6 mg/g depending on cultivation conditions, plant age and tissues [6]. Flavonoids serve as core quality markers for evaluating the medicinal value of *D. huoshanense* [7]. Wild-type *D. huoshanense* resources have been severely depleted due to over-collection and habitat destruction. Secondary-metabolite contents vary greatly among artificially cultivated populations [6]. Therefore, developing low-cost and pollution-free physical quality-improving technologies is of great industrial significance. Many studies have reported plant responses to conventional abiotic stresses such as salinity, drought, and temperature [6,8,9,10]; however, the molecular mechanisms underlying responses to sound- and music-derived mechanical vibrations remain unreported for *D. huoshanense*.

Accumulating evidence demonstrates that sound waves are mechanical-vibration signals perceivable by plants, capable of altering gene expression and secondary-metabolite accumulation [11,12]. Specific sound frequencies promote seedling growth in mung bean and improve total phenolic and vitamin-C contents in tomato [13]. *Oenothera* flowers rapidly increase nectar sugar concentrations within minutes when exposed to pollinator-mimicking sound vibrations [14]. *Eruca sativa* exhibits distinct physiological responses to classical versus rock music, proving that plants can discriminate different acoustic signals [15]. At the molecular level, plant plasma-membrane mechanosensitive ion channels (mid1-complementing activity (MCA), mechanosensitive-like (MSL), piezo mechanosensitive ion channel (PIEZO), osmotic stress-activated calcium-permeable channel (OSCA)) convert mechanical tension caused by sound into intracellular Ca^2+^ signals. Subsequent signal cascades mediated by calmodulin and calcium-dependent protein kinase (CDPK) modulate transcription-factor activities and further alter transcription of downstream metabolic genes [16]. Transcriptomic studies in *Arabidopsis thaliana* have shown that sound vibrations of different frequencies induce divergent sets of differentially expressed genes, change endogenous phytohormone levels (auxin, gibberellin, salicylic acid, abscisic acid), and pre-activate plant defence-related genes [17,18,19].

Music represents complex sound waves organized by multi-dimensional acoustic parameters including pitch, rhythm, tempo, and intensity [20]. Different parameter combinations produce musical signals with distinct stylistic and emotional attributes. Most existing plant-acoustics research uses simple single-frequency pure tones. Comparable transcriptomic investigations on how medicinal plants respond to complex musical signals with distinct acoustic and stylistic features remain scarce [21,22]. Flavonoid-biosynthesis-related enzymes such as chalcone synthase (CHS), hydroxycinnamoyl-CoA shikimate/quinate hydroxycinnamoyl transferase (HCT), and caffeoyl-CoA *O*-methyltransferase (CCoAOMT) largely determine the medicinal quality of *D. huoshanense*, yet their transcriptional responses to musical acoustic stimuli are poorly characterized. Therefore, we performed time-series RNA-seq on *D. huoshanense* plantlets subjected to two representative musical treatments with highly divergent acoustic characteristics. The objectives of this study were as follows: (i) to characterize global transcriptomic shifts triggered by different musical stimuli; (ii) to dissect differential temporal expression patterns of core flavonoid-biosynthetic genes; (iii) to identify hub genes (screened from flavonoid-biosynthetic core seed genes) and candidate transcription factors through co-expression network analysis. This work aims to provide fundamental transcriptomic evidence for exploring music-based physical cultivation techniques, supporting eco-friendly, chemical-free horticultural improvement for this valuable medicinal orchid.

## 2. Materials and Methods

### 2.1. Plant Materials and Growth Conditions

One-year-old acclimatized plantlets of *D*. *huoshanense* were purchased from Yihu Biotechnology Co., Ltd. (Lu’an City, Anhui Province, China). Plantlets were cultivated in growth chambers at 25 °C, 65% relative humidity, and a light intensity of 1950 lux under a 12 h light/12 h dark photoperiod, without any fertilization or irrigation (including spraying). Uniform, healthy, pest-free plantlets were divided into three experimental groups.

### 2.2. Musical-Stimulation Treatments

Three independent incubators with identical hardware and environmental parameters were equipped with loudspeakers to guarantee consistent temperature, humidity, and light conditions across groups: (i) Control group (CK): no music playback; (ii) Forest-music group (Forest): loop-play of Forest Fantasy at 60–70 dB; (iii) Erquan-music group (Erquan): loop-play of the erhu piece Erquan Yingyue at 60–70 dB. The two musical stimuli were defined based on objective acoustic spectral features and stylistic attributes. Forest Fantasy is representative natural-style music with a medium-fast tempo, abundant high-frequency components, rich timbre layers and bright auditory characteristics. By contrast, the erhu solo Erquan Yingyue exhibits a slow tempo and dominant low-frequency components, producing melancholy and restrained auditory perception. To match the regular growth rhythm of plantlets, a standardized acoustic stimulation regime was strictly implemented: Music was played continuously for 12 h during the light period and terminated during the 12 h dark period. Samples at 1, 6, 12 h corresponded to the musical stimulation stage, while the 24 h time point belonged to the dark recovery stage without acoustic treatment. The CK group represents time-matched controls: leaf samples from non-stimulated plantlets were harvested synchronously at each time point, parallel to sample collection of the two music-treatment groups. Leaf samples were collected at 1 h, 6 h, 12 h, and 24 h after the initiation of musical treatment. Specifically, the 1 h, 6 h, and 12 h time points corresponded to the continuous music and light treatment stage, while the 24 h time point corresponded to the dark recovery stage without music stimulation. For each time point and each treatment group, six uniform and healthy seedlings were randomly selected and mixed to form a single biological replicate, and three independent biological replicates were prepared for all groups. All collected samples were immediately snap-frozen in liquid nitrogen and stored in an ultra-low-temperature refrigerator at −80 °C for subsequent total RNA extraction and transcriptome sequencing. Ultimately, a total of 36 valid sequencing samples were obtained (3 treatments × 4 time points × 3 biological replicates).

### 2.3. RNA Extraction, Library Construction, and High-Throughput Sequencing

Total RNA was extracted using the magnetic-bead-based plant total-RNA kit DP762 (Tiangen, Beijing, China). RNA concentration and purity were assessed using a Nanodrop 2000 spectrophotometer (Thermo Fisher Scientific, Waltham, MA, USA); RNA integrity was checked with an Agilent 5400 system (Agilent Technologies, Santa Clara, CA, USA), with the acceptance threshold RIN ≥ 6.8. Poly(A)-mRNA was enriched by oligo-dT magnetic beads, fragmented, and used for first- and second-strand cDNA synthesis with random hexamer primers. Double-stranded cDNA was purified, end-repaired, A-tailed, and ligated to Y-shaped sequencing adapters. After 15 cycles of PCR amplification, target fragments were recovered by agarose-gel electrophoresis for library construction. PE150 paired-end sequencing was performed on the NovaSeq 6000 platform (Illumina, San Diego, CA, USA) by Sanshu Biotechnology (Nantong, China) and Bio&Data Biotechnology (Guangzhou, China).

### 2.4. Sequencing-Data Quality Control and Gene-Function Annotation

Raw sequencing reads were processed with fastp (version 0.23.4) to remove adapters and low-quality reads to obtain clean reads [23]. Clean reads were mapped against the chromosome-scale *D*. *huoshanense* reference genome (accession CNA0014590) using STAR 16.0 software [24]. Read counts per gene were calculated by featureCounts for gene-expression quantification. Gene-function annotation was conducted by DIAMOND v2.2.x against NR, Swiss-Prot, KOG, GO, and KEGG databases [25]. Transcription-factor families were annotated based on the PlantTFDB database [26].

### 2.5. Differential-Expression-Gene Screening

Differential-expression analysis was performed using the DESeq2 R package [27]. DEG identification was conducted within each individual time point: at each sampling moment (1 h, 6 h, 12 h, 24 h), two pairwise comparisons were executed: Forest group versus time-matched CK, and Erquan group versus time-matched CK. Genes satisfying (|log_2_FC| > 1) and adjusted *p*-value (*padj*) < 0.05 were defined as differentially expressed genes (DEGs). Gene-expression values were further normalized to FPKM to eliminate biases caused by gene length and sequencing library size. Note that FPKM-normalized values were adopted for downstream visualizations and correlation-based analyses, including sample-correlation heatmap, PCA, expression-profile heatmaps and Pearson-correlation calculation for co-expression network construction. Gene expression values were normalized to FPKM. GO and KEGG enrichment analyses were implemented via clusterProfiler [28]; Fisher’s exact test with Benjamini–Hochberg correction was specifically used for GO/KEGG enrichment to control the false-discovery rate; FDR < 0.05 was regarded as statistically significant enrichment.

### 2.6. Sample Correlation, Principal-Component Analysis, and Expression Clustering

Pearson correlation coefficients among all samples were calculated based on log_2_(FPKM + 1) normalized expression values for correlation-heatmap visualization. Gene-expression matrices were z-score normalized for PCA. The top 2000 most variable genes were subjected to hierarchical clustering using Euclidean distance and average linkage to generate a global expression heatmap, to evaluate biological reproducibility and experimental grouping validity.

### 2.7. Expression Analysis of Flavonoid-Pathway Genes

Annotated genes belonging to ko00941 (flavonoid biosynthesis) and ko00944 (flavone and flavonol biosynthesis) were extracted. Two ratios were calculated: log_2_(treatment mean/corresponding CK mean) and log_2_(Forest mean/Erquan mean). Row-clustered temporal heatmaps were plotted. Core pathway enzymes were divided into Class A and Class B modules according to their expression patterns under the two musical treatments. Class A genes correspond to the expression pattern of Forest_up/Erquan_ns-or-down: significantly up-regulated under Forest-music treatment, while non-significant or repressed under Erquan-music treatment. By contrast, Class B genes follow the pattern of Erquan_down/Forest_ns-or-up: significantly down-regulated under Erquan-music treatment, while non-significant or unchanged under Forest-music treatment.

### 2.8. Upset Analysis of Time-Point-Specific Sustained DEGs

Upset plots were generated for up- and down-regulated DEGs from Forest and Erquan groups across four time points. Core DEGs: genes showing significant differential expression at all four time points. Sustained DEGs: genes showing significant differential expression at ≥3 time points. Two specific sustained-DEG sets were further defined: Set 1: Forest significantly up-regulated at ≥3 s while Erquan up-regulated at ≤1 time point (60 genes, Forest-specific sustained up-regulated genes). Set 2: Erquan significantly down-regulated at ≥3 time points while Forest down-regulated at ≤1 time point (74 genes, Erquan-specific sustained down-regulated genes). KEGG enrichment was performed for both gene sets.

### 2.9. qRT-PCR Analysis

Six representative DEGs from Class A and Class B flavonoid-biosynthesis modules were selected for qRT-PCR. qRT-PCR was performed on a LightCycler^®^ 96 Instrument (Roche Diagnostics, Mannheim, Germany) using the ChamQ Blue Universal SYBR qPCR Master Mix (Vazyme Biotech, Nanjing, Jiangsu, China, Cat. No. Q312-02). Each 20 μL reaction mixture contained 10.0 μL of 2 × Master Mix, 0.4 μL of forward primer (10 μM), 0.4 μL of reverse primer (10 μM), and 2.0 μL of template cDNA, with ddH_2_O added to reach the final volume. The thermal cycling conditions were as follows: initial denaturation at 95 °C for 5 min; 40 cycles of 95 °C for 10 s and 60 °C for 30 s; followed by melting-curve analysis, which consisted of 95 °C for 15 s, 60 °C for 60 s, and 95 °C for 15 s with continuous fluorescence acquisition. All gene-specific primer sequences are listed in Table S1. *TBP* was used as the reference gene [29]. Three technical replicates were set for each sample. Relative gene expression was calculated using the 2^−ΔΔCt^ method. Expression trends obtained from qRT-PCR were compared against RNA-seq results to assess transcriptome reliability.

### 2.10. Co-Expression-Network Construction

Candidate flavonoid core genes were filtered with the criterion log_2_(Forest/Erquan) > 0 for at least three time points (either satisfied at all four time points or continuously increasing with values > 0 at the last two time points). These core genes refer to flavonoid-biosynthesis-related seed genes derived from prior functional knowledge and KEGG pathway annotation. Candidate core flavonoid genes were combined with the full set of DEGs. Pearson correlation coefficients were computed from log_2_(FPKM + 1) values across all 36 samples; only pairs with (|r| ≥ 0.8) were retained for network construction. After network generation, hub genes were identified according to a network topological feature: node degree (the number of directly connected neighbouring nodes) was calculated for each core seed gene. Genes with top-ranked node-degree values were defined as hub genes in this co-expression network.

### 2.11. Statistical Analysis

All data processing and plotting were performed in R (version 4.3.1). Student’s *t*-test was used for group-wise gene-expression comparisons; *p* < 0.05 was considered statistically significant.

## 3. Results

### 3.1. Sequencing-Data Quality Control and Genome-Annotation Statistics

Clean-read quantities were balanced across treatments: CK (3.47 × 10^7^–5.80 × 10^7^), Forest (3.51 × 10^7^–5.04 × 10^7^), Erquan (3.57 × 10^7^–5.04 × 10^7^). Base quality was high: Q20 ranged from 99.65% to 99.74%, and the minimum Q30 value reached 97.74%, well above general transcriptome-sequencing standards. GC contents varied between 45.31% and 46.27%, without obvious sequencing bias. Total genome-mapping rates were 96.37–96.89%, and unique-mapping rates were 87.44–88.28%, indicating reliable read-alignment and quantitative performance (Figure 1A). In total, 20,247 annotated genomic genes were analyzed: 20,241 annotated in NR (99.97%), 14,564 in PFAMs (71.93%), 10,011 in Swiss-Prot (49.44%), 8853 in GO (43.72%), and 8363 annotated as transcription factors (41.30%). A total of 5405 genes obtained KEGG annotations (26.70%), providing sufficient background for subsequent pathway-enrichment analysis (Figure 1B).

### 3.2. Sample Reproducibility and Global Transcriptome Separation

Pearson correlation coefficients among biological replicates were all higher than 0.94, demonstrating good experimental reproducibility (Figure S1). PCA showed that PC1 explained 20.6% of total variance and PC2 explained 16.2% of total variance. CK, Forest, and Erquan samples were clearly separated in two-dimensional space and clustered according to treatment type and sampling time (Figure S2). Hierarchical clustering of the top-2000 most variable genes agreed with PCA results. Musical-treatment type and treatment duration were the main drivers of transcriptome divergence, confirming valid experimental grouping (Figure 2, Table S2).

### 3.3. Global Characteristics of Differentially Expressed Genes

Across eight treatment-versus-control comparisons, both musical treatments reached their DEG count peak at 12 h. Forest-12 h produced 1271 DEGs (489 up-regulated, 782 down-regulated); Erquan-12 h produced 1168 DEGs (294 up-regulated, 874 down-regulated). The proportion of down-regulated genes was higher under Erquan treatment (Table S2). KEGG enrichment exhibited treatment-specific patterns. The phenylpropanoid and flavonoid-biosynthesis pathways were significantly enriched in Forest-1 h vs. CK (Figure 3A). The flavonoid-biosynthesis pathway achieved the highest enrichment significance in this study for Erquan-24 h vs. CK (*padj* = 1.96 × 10^−5^) (Figure 3B).

### 3.4. Expression Patterns of Two Classes of Flavonoid-Biosynthesis Genes

Among 38 annotated genes in ko00941 and ko00944, 23 genes were stably expressed across all samples (Table S3). According to temporal-heatmap expression behaviour, core enzymes in the flavonoid-biosynthesis pathway were divided into Class A and Class B (Table S4). Class A genes (*CHS*, *NOMT*) were significantly up-regulated only at 1 h and 6 h under Forest treatment, while unchanged or repressed under Erquan. Class B genes (*F3H*, *F3′H*, *HCT*, *CCoAOMT*) were persistently down-regulated at multiple time points under Erquan but remained stable under Forest. Notably, HCT was down-regulated at all four time points in the Erquan group (Figure 4, Table 1).

### 3.5. Time-Point-Specific Sustained Differentially Expressed Genes

Upset-plot analysis revealed that DEGs differing at all four time points were limited in number: Forest sustained-up = 22, sustained-down = 11 (Figure 5A,B); Erquan sustained-up = 8, sustained-down = 23 (Figure 5C,D). These small sets were insufficient for reliable enrichment. Therefore, genes showing significant differences at ≥3 time points were defined as sustained DEGs. The 60 Forest-specific sustained up-regulated genes were significantly enriched in purine metabolism (FDR = 0.019; Figure 5E). The 74 Erquan-specific sustained down-regulated genes were significantly enriched in flavonoid biosynthesis (FDR = 0.038; Figure 5F).

### 3.6. qRT-PCR Validation

To evaluate transcriptome reliability, six representative structural genes belonging to Class A and Class B flavonoid-biosynthesis modules were selected for qRT-PCR: *CHS* (Dhu000016901), *NOMT* (Dhu000027926), *F3H* (Dhu000002865), *F3′H* (Dhu000012876), *HCT* (Dhu000022982), and *CCoAOMT* (Dhu000026789). Relative expression was normalized against the *TBP* reference gene [29]. The up-/down-regulation directions obtained by qRT-PCR were generally consistent with FPKM-based RNA-seq trends, despite minor quantitative amplitude deviations in individual samples. This result confirmed that our transcriptome dataset is trustworthy for downstream analyses such as co-expression-network construction (Figure 6).

### 3.7. Co-Expression Network of Core Flavonoid-Biosynthesis Genes

Thirteen candidate flavonoid core genes covering skeleton-biosynthesis, hydroxylation, and methylation enzymes were retained for network construction (Table S5). Under the threshold (|r| ≥ 0.8), 266 significant co-expression pairs (229 positive, 37 negative) were obtained, comprising 250 network genes (Figure S4). To improve visualization readability, subnetworks centred on three major hub genes (*C4H*, *F3′H*, and *CCoAOMT*) with their directly connected neighbours are presented in Figure 7A–C. Hub structural genes (*C4H*, *F3′H*, *CCoAOMT*) were screened from flavonoid-biosynthetic core seed genes based on node-degree topological metrics of the co-expression network. *C4H* possessed the largest number of co-expression partners (117), followed by *F3′H* (58) and *CCoAOMT* (31) (Table S6). Twenty-eight transcription-factor genes from 18 families were embedded within the network; WRKY (6), MYB (3), and NAC (3) represented the most abundant families (Table S7). KEGG enrichment of all 250 network genes showed that flavonoid biosynthesis attained the highest significance (15 genes, FDR = 6.40 × 10^−15^), followed by stilbenoid, diarylheptanoid, gingerol biosynthesis, phenylpropanoid biosynthesis, and α-linolenic-acid metabolism (Figure 7D).

## 4. Discussion

Using time-series transcriptomics, the present study dissected molecular responses of *D*. *huoshanense* to two musical stimuli with contrasting stylistic features. Multiple lines of evidence, including DEG enrichment, gene-module classification, treatment-specific sustained-DEG sets, and co-expression networks, collectively demonstrated that natural-style music and melancholy-style music trigger opposite transcriptional reprogramming of flavonoid-biosynthesis pathways.

Plant plasma-membrane mechanosensitive ion channels (MSICs) rapidly convert mechanical vibration into intracellular Ca^2+^ secondary-messenger signals within minutes to tens of minutes [30]. Calcium-signal bursts further activate downstream transcription-factor cascades and drive transcriptional reprogramming of flavonoid-biosynthesis-related genes. The rapid up-regulation of *CHS* and *NOMT* [31,32] as early as 1 h under Forest treatment can be plausibly interpreted through this MSIC-mediated Ca^2+^-signalling framework, although further functional experiments are still required for direct verification. Previous work has demonstrated that sound stimuli of particular frequencies and intensities can reshape plant secondary-metabolite accumulation within hours to days [33,34]. For instance, single-frequency sound waves and complex musical signals with divergent frequency spectra, usually applied at 60–80 dB intensity, have been reported to modulate the biosynthesis of phenolics, vitamin C, flavonoids and stilbenoids in multiple plant species [17,33,34]. Nevertheless, acoustic-signal effects on phenylpropanoid pathways are highly context-dependent and species-specific [35]. In our study, slow-tempo, low-frequency musical signals from Erquan Yingyue activated stress-like transcriptional programs supported by multiple transcriptomic observations: a far larger number of genes were repressed than induced under Erquan treatment (874 down-regulated versus 294 up-regulated DEGs at 12 h); Erquan-specific sustained-down-regulated genes were significantly enriched in stress-relevant pathways including plant-pathogen interaction and plant hormone signal transduction [17,36]. However, this stress response failed to induce canonical defence-oriented flavonoid accumulation; instead, multiple late-modification flavonoid genes were transcriptionally repressed. Under classic abiotic stresses such as drought or UV-B irradiation, plants usually boost flavonoid biosynthesis for antioxidative defence [37,38]. Therefore, the stress-like transcriptomic signature triggered by this melancholy musical stimulus represents a distinctive transcriptional output different from conventional abiotic-stress responses. When plants endure persistent adverse stimuli, growth-defence resource trade-offs may occur; under severe pressure secondary-metabolite pathways can even be suppressed to prioritize basic survival [39,40]. This resource-reallocation hypothesis remains to be validated by combined time-series transcriptome-metabolome datasets.

Our transcriptomic evidence illustrated that natural-style acoustic treatment from Forest Fantasy could rapidly activate upstream flavonoid biosynthetic genes within 1–6 h, which provides a practical hint for horticultural production. Short-period natural-style musical stimulation applied at the seedling stage may serve as a chemical-free physical measure to potentially enhance flavonoid-related medicinal value of *D. huoshanense*. In contrast, prolonged exposure to slow-tempo, low-frequency musical signals may induce a stress-like transcriptional response and inhibit flavonoid modification-related genes in multiple late steps. It should be noted that our transcriptome data only captured early-phase molecular responses within 24 h of acoustic treatment in seedling-stage plantlets. This observation implies that careful selection of acoustic materials is required when deploying musical stimulation in medicinal-orchid horticultural practice. Temporal transcriptomic patterns revealed divergent timing of action for the two musical treatments: Forest exerted transient induction effects mainly within 1–6 h, while Erquan produced sustained transcriptional-suppression effects lasting up to 24 h. Enrichment results for treatment-specific sustained DEGs further indicated activation of distinct transcriptional cascades. Forest-specific sustained up-regulated genes were enriched in purine metabolism, suggesting concurrent modulation of nucleotide- and energy-related metabolism besides secondary metabolism; Erquan-specific sustained down-regulated genes concentrated on flavonoid-biosynthesis pathways.

Co-expression-network analysis identified *C4H*, *F3′H*, and *CCoAOMT* as core genes co-expressed with MYB, WRKY, and NAC transcription-factor candidates. MYB proteins are well-established master regulators of plant flavonoid biosynthesis [41]; WRKY and NAC families are central integrators of diverse abiotic-stress signals [42,43]. It must be emphasized that our co-expression network only reflects statistical expression-level correlations and cannot confirm direct protein-DNA physical interactions. Candidate TF–structural gene modules uncovered here require future in vivo functional assays such as yeast-one-hybrid and transient-overexpression experiments.

From an application perspective, our findings propose new ideas for pollution-free quality-improvement cultivation of *D. huoshanense*. Short-term natural-style musical playback during seedling propagation may activate flavonoid-biosynthesis-related transcription without exogenous chemical inputs, matching the requirements of green medicinal-plant production. Meanwhile, this study expands our understanding of plant acoustoperception: plants can perceive not only simple single-frequency sound waves but also complex multi-parameter musical–acoustic signals with divergent stylistic characteristics.

Several important limitations of this study should be acknowledged. First, the current conclusions are merely supported by transcriptomic and qRT-PCR evidence, while corresponding metabolite quantification and gene functional validation are still lacking. Second, only two typical musical styles were adopted in this study. We cannot confirm whether other music tracks with similar tempo-frequency features could reproduce the observed transcriptomic responses. Testing multiple independent audio materials per style in further experiments will help exclude piece-specific effects and verify style-dependent biological outputs. Therefore, more types of acoustic stimuli are required to generalize the universal regulatory patterns of sound-mediated flavonoid metabolism. Third, these two representative pieces were selected for three reasons: (i) they mimic two distinct complex acoustic patterns existing in plant ambient environments, complementing previous studies focusing mainly on single-frequency pure tones; (ii) all experimental confounding variables including sound loudness, playback rhythm, hardware facilities and plant growth conditions were strictly unified across treatments; only the musical–acoustic characteristics differ, making pairwise comparison feasible; (iii) both musical works are publicly available, facilitating experimental repeatability by follow-up researchers. Although experimental conditions were well-controlled, we cannot rule out potential piece-specific effects, as mentioned above. Fourth, only a single 12 h light-phase music-exposure regime was applied in this work. This duration was chosen to match the 12 h photoperiod of *D. huoshanense* plantlets, enabling us to capture successive transcriptomic events covering early rapid responses (1 h, 6 h), mid-term sustained responses (12 h), and post-stimulus dark-recovery responses (24 h). Our time-series data showed obvious gene reprogramming as early as 1 h and 6 h, yet the maximum number of DEGs appeared at 12 h, suggesting that 12 h continuous light-phase stimulation could capture comprehensive transcriptional alterations of flavonoid-biosynthesis pathways under our experimental setup. Nevertheless, we lack shorter-exposure gradient groups to resolve the minimal effective stimulation duration triggering flavonoid-related transcriptional reprogramming. Multi-duration gradient experiments should be carried out in future work. Fifth, the complete signal transduction cascade underlying plant acoustic perception remains to be further elucidated. Future work will integrate widely targeted metabolomics and genetic functional assays to systematically clarify the molecular mechanism of acoustic-regulated flavonoid biosynthesis, and further optimize sound-assisted green cultivation strategies for high-quality production of medicinal orchids.

## 5. Conclusions

Time-series transcriptomic analysis demonstrated that *D*. *huoshanense* undergoes divergent transcriptional reprogramming of flavonoid biosynthesis when exposed to musical acoustic stimuli of contrasting styles. Natural-style Forest Fantasy rapidly up-regulated early flavonoid-biosynthetic genes (*CHS*, *NOMT*) within 1–6 h, while melancholy-style Erquan Yingyue persistently repressed multiple late-step modification genes (*F3H*, *F3′H*, *HCT*, *CCoAOMT*). Co-expression network analysis pinpointed *C4H*, *F3′H*, and *CCoAOMT* as hub structural genes co-expressed with candidate MYB, WRKY and NAC transcription factors. Our findings suggest that short-duration natural-style acoustic stimulation represents a promising chemical-free horticultural approach for quality improvement of *D*. *huoshanense*. Additional metabolome measurements and gene-function validations are required in future work to verify the practical efficacy of musical–acoustic treatments in real-world horticultural production.

## Figures and Tables

**Figure 1:**
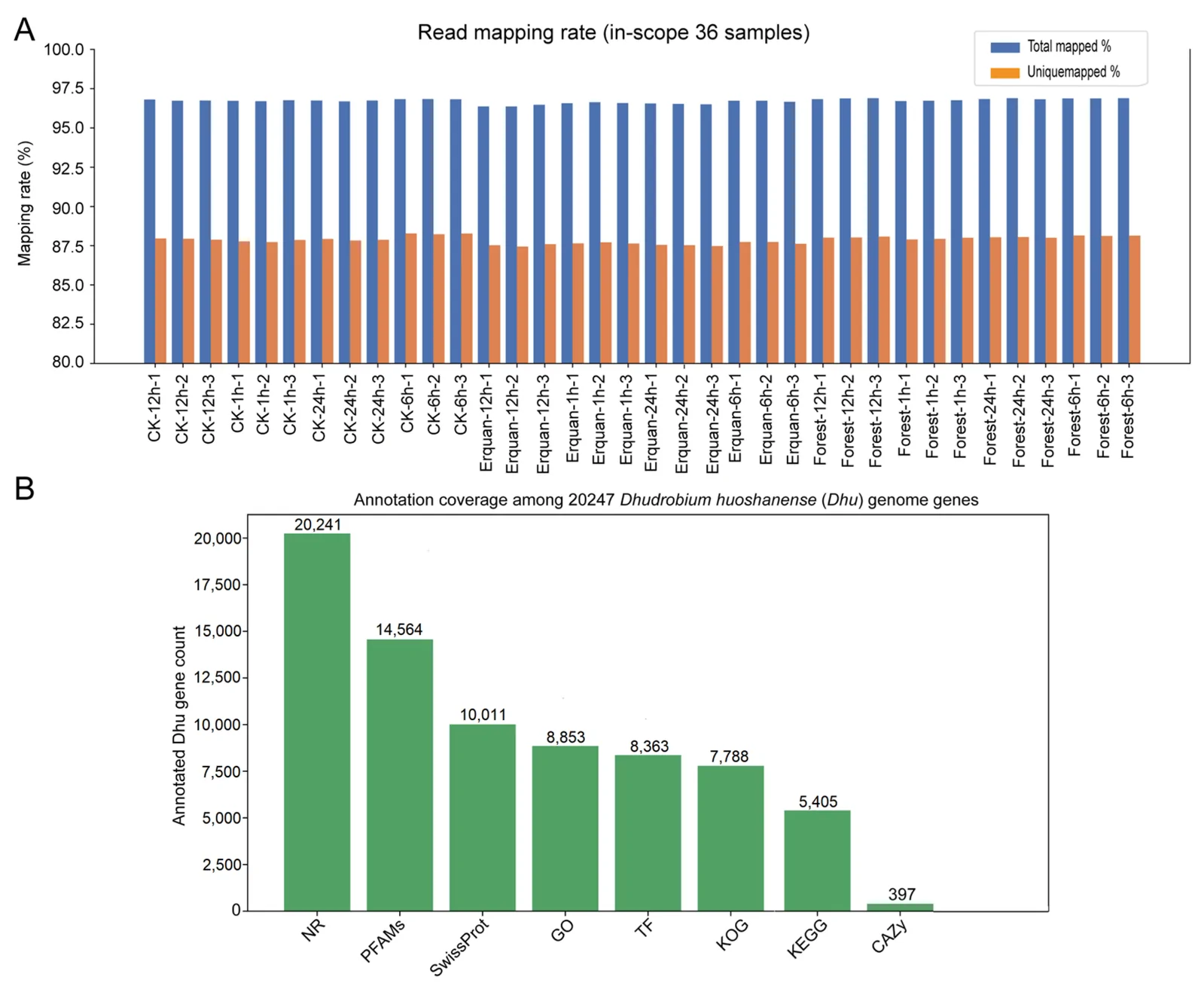
Transcriptome sequencing quality and gene functional annotation. (**A**) Read mapping statistics of 36 sequencing samples. Blue and orange bars indicate total and unique mapping rates against the *D*. *huoshanense* reference genome, respectively. All samples showed high alignment quality with stable mapping rates. (**B**) Gene annotation profiles across functional databases. In total, 20,247 genes were annotated in multiple databases, and 5405 genes were annotated in the KEGG database, supporting subsequent pathway enrichment analysis.

**Figure 2:**
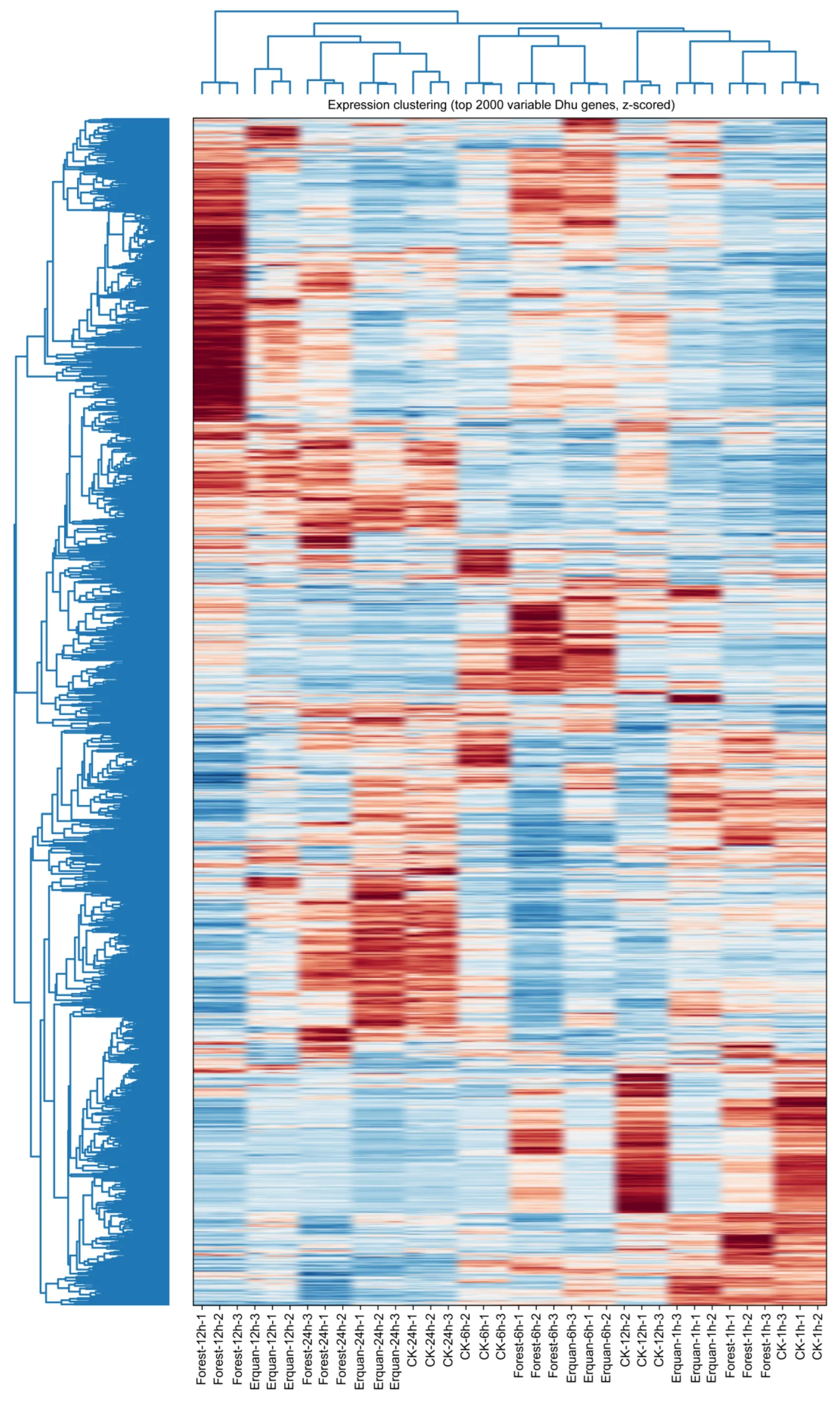
Hierarchical clustering heatmap of the top 2000 variable genes with z-score normalization. Columns stand for samples of CK, Forest and Erquan groups at four time points. Red = high expression, blue = low expression. Sample clustering based on treatment and time gradients matched PCA results, confirming valid experimental grouping.

**Figure 3:**
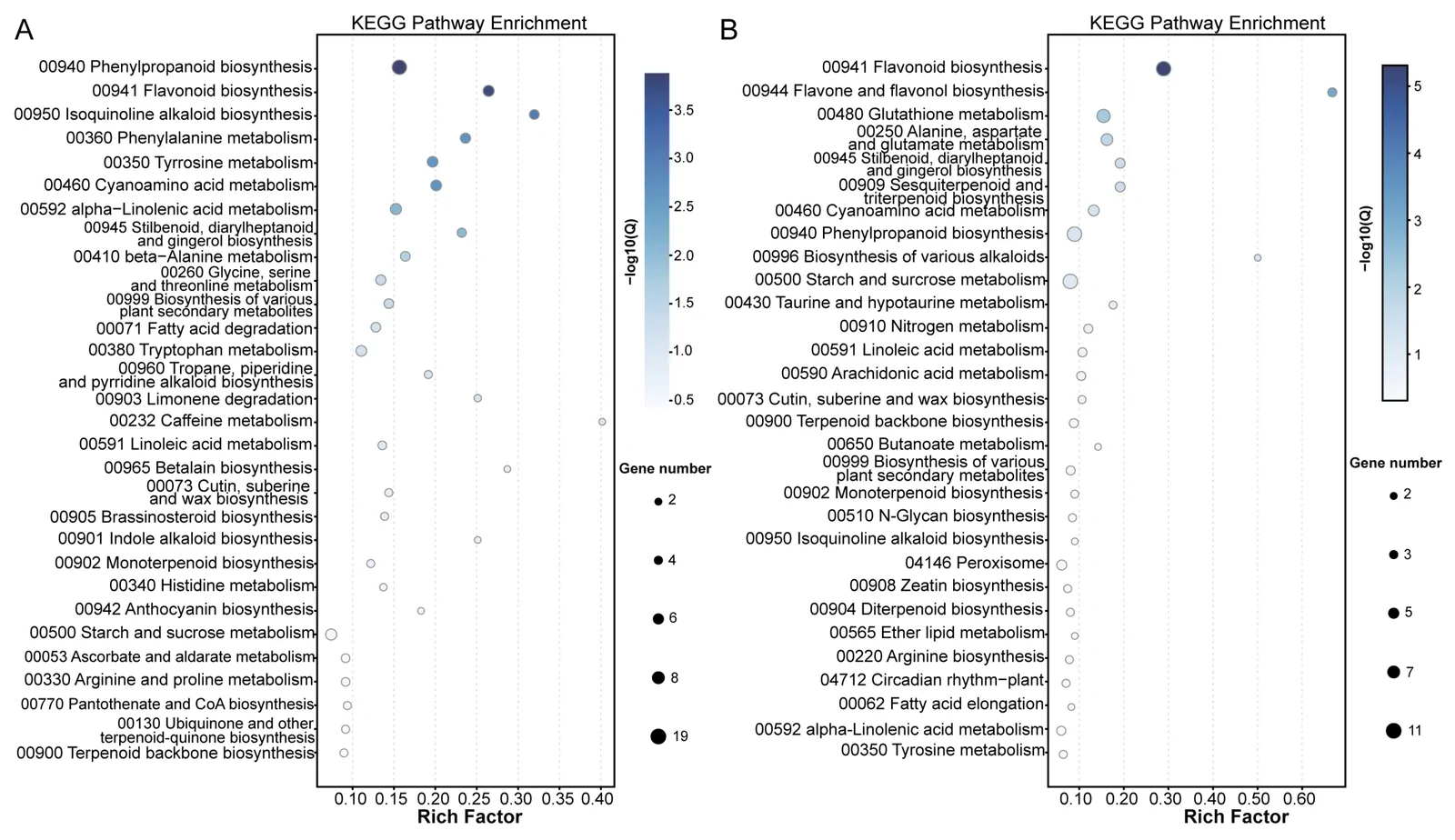
KEGG enrichment bubble plots for DEGs. (**A**) Forest-1 h vs. CK; (**B**) Erquan-24 h vs. CK. X-axis: Rich factor; bubble colour: adjusted Q-value (FDR); bubble size: number of enriched DEGs. Flavonoid-biosynthesis-related pathways exhibited significant enrichment in both datasets.

**Figure 4:**
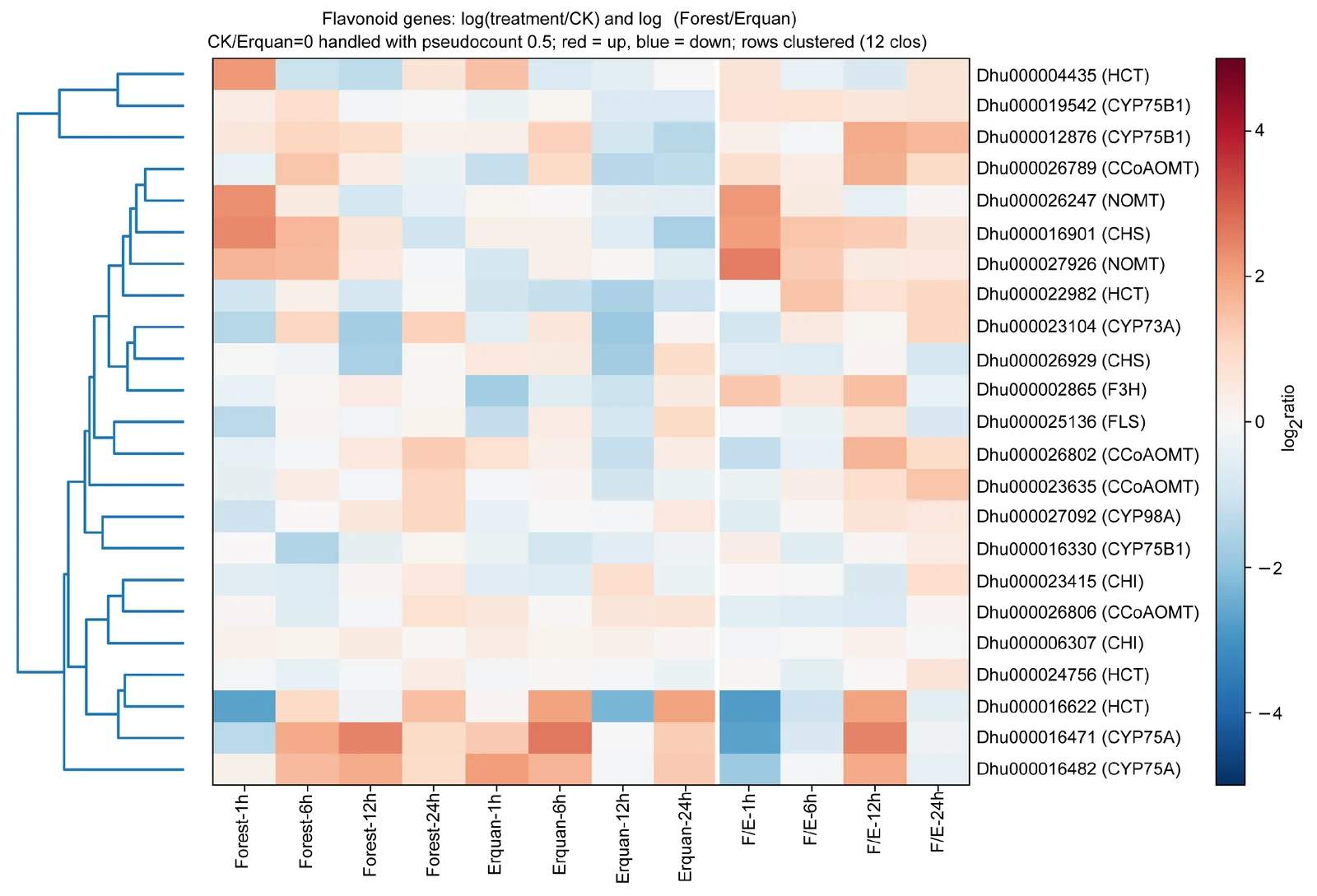
Temporal expression profiles of genes involved in flavonoid biosynthesis (ko00941) and flavone and flavonol biosynthesis (ko00944) in *D*. *huoshanense* under different musical–acoustic stimuli. Gene expression changes were quantified as log_2_-transformed ratios of treatment/CK and Forest/Erquan at four sampling time points. Row-wise clustering was performed, with red and blue colours representing up-regulated and down-regulated expression patterns, respectively.

**Figure 5:**
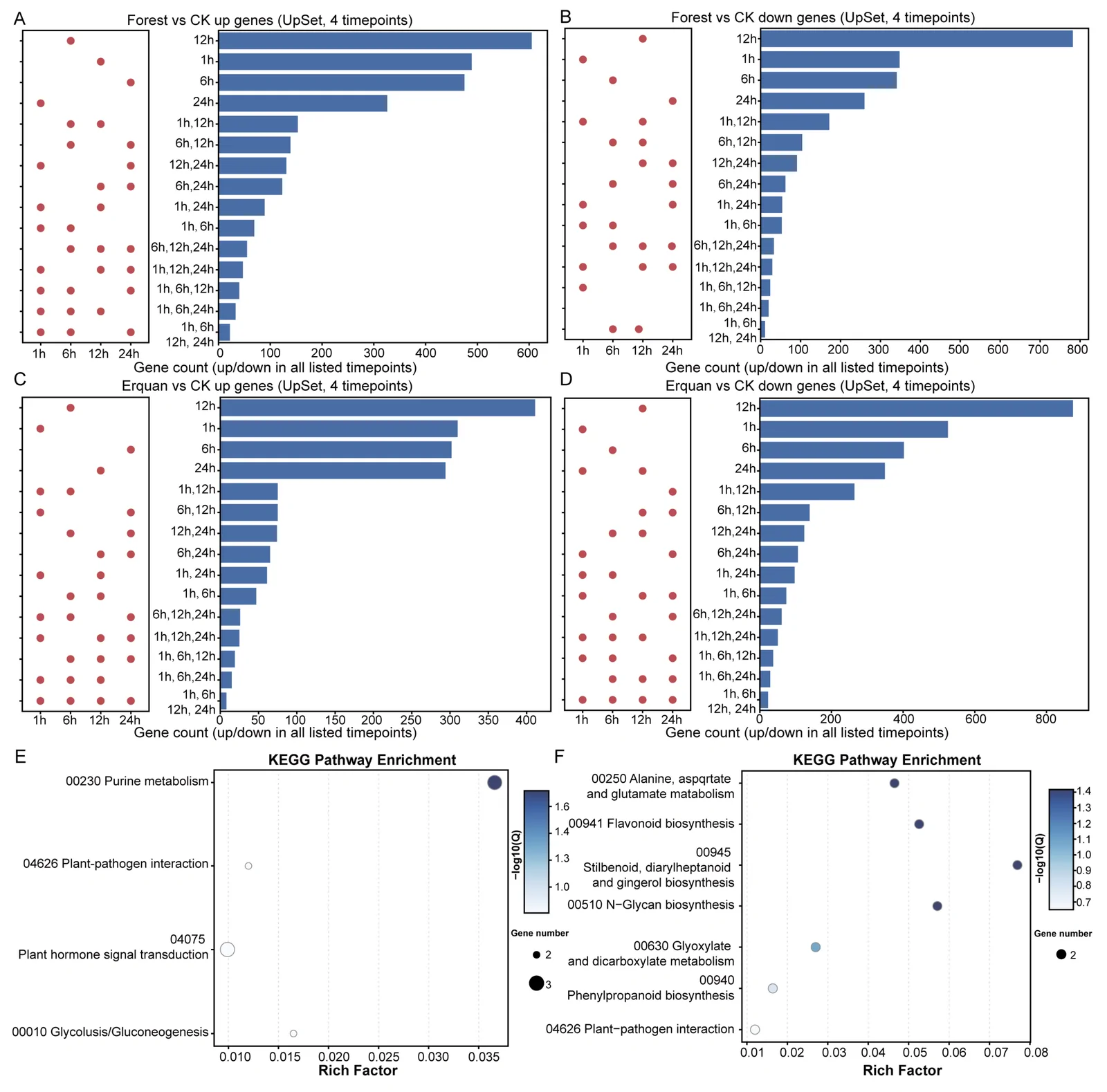
Time-series differential gene overlap and KEGG enrichment analysis of treatment-specific sustained DEGs induced by two musical stimuli. (**A**–**D**) UpSet plots showing overlaps of up-regulated and down-regulated DEGs across four time points (1, 6, 12, 24 h) for Forest and Erquan groups, respectively. (**A**) Up-regulated DEGs of Forest vs. CK; (**B**) Down-regulated DEGs of Forest vs. CK; (**C**) Up-regulated DEGs of Erquan vs. CK; (**D**) Down-regulated DEGs of Erquan vs. CK. Dots on the left denote combinations of sampling time points, and horizontal bars represent the number of intersecting DEGs in each set. (**E**) KEGG enrichment bubble plot for Forest-specific sustained up-regulated genes (60 genes); the top enriched pathway is purine metabolism (map00230, FDR = 0.019). (**F**) KEGG enrichment bubble plot for Erquan-specific sustained down-regulated genes (74 genes); flavonoid biosynthesis (map00941) exhibited significant enrichment (FDR = 0.038). Bubble size corresponds to the number of enriched genes within each pathway, and colour gradient reflects adjusted FDR (Q-value). Rich factor is defined as the ratio of enriched gene count to total background genes in a given pathway.

**Figure 6:**
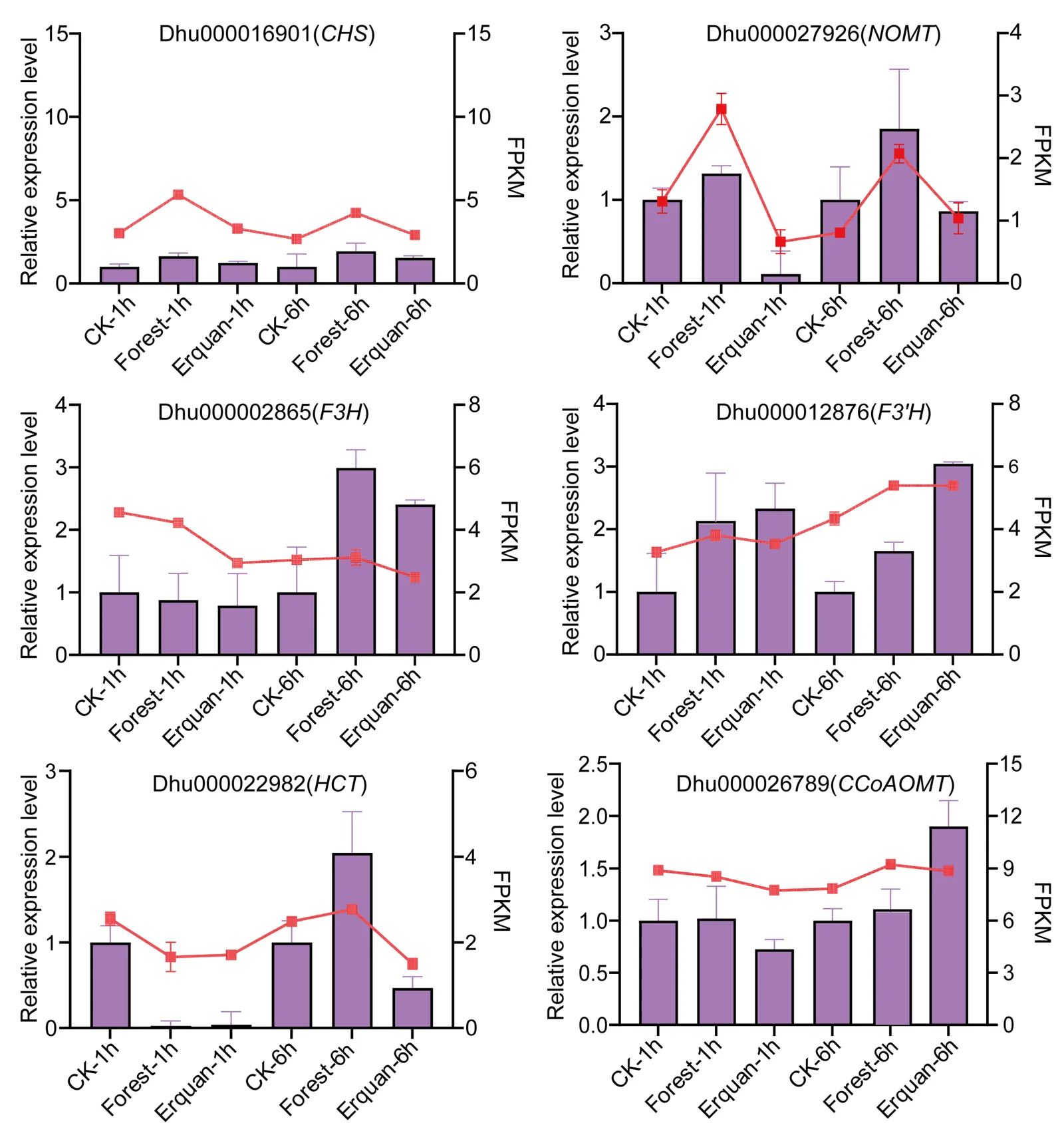
qRT-PCR validation of six flavonoid-biosynthetic genes. Purple bars: relative expression from qRT-PCR; red lines: FPKM values from RNA-seq. Wilcoxon signed-rank tests were performed to assess whether relative expression differed from the CK-1h baseline value of 1. Pairwise correlation between normalized qRT-PCR and RNA-seq data is presented in Figure S3. Expression trends are largely concordant between the two datasets, confirming transcriptome reliability.

**Figure 7:**
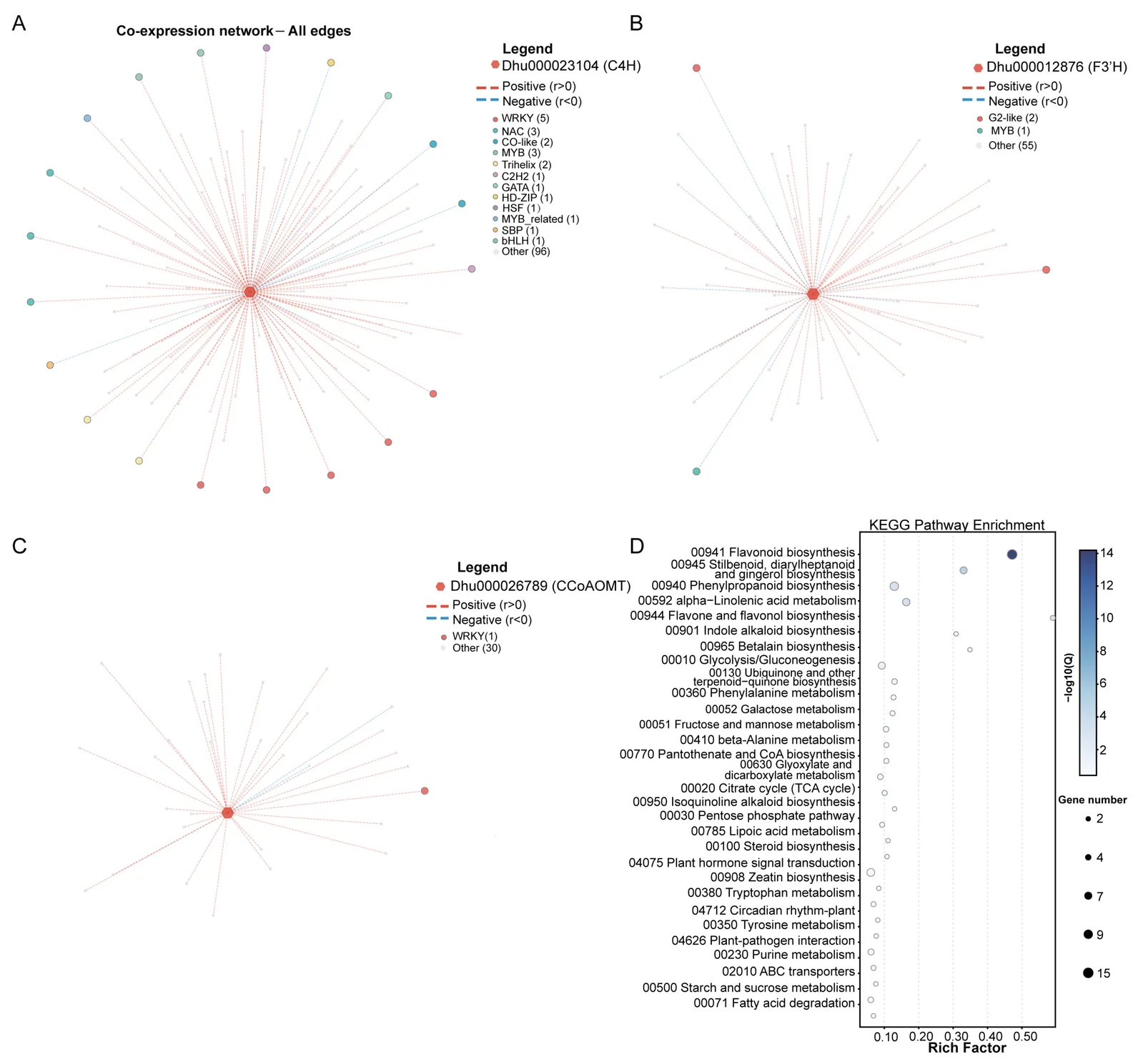
Co-expression sub-network analysis and KEGG pathway enrichment of selected flavonoid-biosynthesis-related core genes in *D. huoshanense*. (**A**–**C**) Gene co-expression subnetworks centred on three core flavonoid biosynthetic genes: Dhu00023104 (*C4H*), Dhu00012876 (*F3′H*), and Dhu00026789 (*CCoAOMT*), respectively. Red dashed lines indicate positive co-expression correlations (r > 0), while blue solid lines represent negative co-expression correlations (r < 0). The surrounding nodes correspond to functionally classified co-expressed partner genes (transcription factors and other functional genes), and different colours of peripheral nodes denote distinct functional categories of the interacting genes. (**D**) KEGG enrichment bubble chart for network-included genes. X-axis = Rich factor; colour = Q-value; bubble size = enriched-gene count. Flavonoid biosynthesis was the top-enriched pathway. Core flavonoid genes were placed in the network centre; transcription factors were arranged in outer nodes coloured by family. KEGG enrichment was carried out for all genes within the network.

**Table 1:** Two groups of flavonoid biosynthesis genes were selected based on differential expression under forest and Erquan stimuli.

Category	Gene ID	Gene Full Name & Abbreviation	Expression Regulatory Pattern Across Treatments	*p*-Value	Fold Change
Class A	Dhu000016901	chalcone synthase (*CHS*)	Significantly up-regulated at 1 h and 6 h under Forest treatment; unchanged or repressed upon Erquan stimulation	F-1 h: 1.34 × 10^−231^ F-6 h: 6.46 × 10^−69^	F-1 h: 5.39 F-6 h: 3.40
Class A	Dhu000027926	naringenin 7-*O*-methyltransferase (*NOMT*)	Markedly induced at 1 h and 6 h by Forest music; down-regulated at 1 h and 24 h under Erquan exposure	F-1 h: 3.40 × 10^−14^ F-6 h: 2.75 × 10^−10^ E-1 h: 3.52 × 10^−3^ E-24 h: 1.03 × 10^−1^	F-1 h: 3.88 F-6 h: 4.37 E-1 h: 0.38 E-24 h: 0.41
Class B	Dhu000002865	naringenin 3-dioxygenase (*F3H*)	Continuously down-regulated at 1 h and 12 h with Erquan treatment; no obvious expression change in Forest group	E-1 h: 3.50 × 10^−55^ E-12 h: 9.98 × 10^−12^	E-1 h: 0.28 E-12 h: 0.43
Class B	Dhu000012876	flavonoid 3′-monooxygenase (*F3′H*)	Repressed at 12 h and 24 h after Erquan stimulation; stable expression under Forest acoustic stimuli	E-12 h: 3.12 × 10^−23^ E-24 h: 3.44 × 10^−25^	E-12 h: 0.50 E-24 h: 0.35
Class B	Dhu000022982	Phenylpropanoid biosynthesis (*HCT*)	Persistently down-regulated at all four time points (1/6/12/24 h) in Erquan group; no repression in Forest group	E-1 h: 1.61 × 10^−07^ E-6 h: 9.83 × 10^−09^ E-12 h: 8.38 × 10^−20^ E-24 h: 3.72 × 1^0−07^	E-1 h: 0.45 E-6 h: 0.39 E-12 h: 0.27 E-24 h: 0.42
Class B	Dhu000026789	Caffeoyl-CoA *O*-methyltransferase (*CCoAOMT*)	Significantly down-regulated at 1 h, 12 h and 24 h with Erquan treatment; unaffected by Forest music	E-1 h: 3.27 × 10^−267^ E-12 h: 6.68 × 10^−205^ E-24 h: 2.68 × 10^−194^	E-1 h: 0.43 E-12 h: 0.37 E-24 h: 0.42

## Data Availability

The data presented in this study are openly available in GenBank under BioProject accession PRJNA1518869, and the raw sequence reads are deposited in the Sequence Read Archive (SRA) under accessions SRR40350549–SRR40350584.

## Supplementary Materials

The following supporting information can be downloaded at: https://www.mdpi.com/article/10.3390/genes17101178/s1, Table S1: List of primers used in this study for qRT-PCR. Table S2: Summary of the number of differentially expressed genes (DEGs) across eight comparison groups. Table S3: FPKM expression values of 23 stably expressed genes involved in flavonoid biosynthesis (ko00941) and flavone and flavonol biosynthesis (ko00944) across all 36 RNA-seq samples. Table S4: Classification of 23 flavonoid-biosynthesis genes into two regulatory categories based on their expression patterns under Forest and Erquan musical–acoustic treatments. Table S5: Thirteen candidate flavonoid-biosynthetic genes screened by Forest/Erquan expression ratio. Table S6: Number of co-expression neighbors for 13 candidate flavonoid biosynthetic core seed genes. Table S7: Summary of transcription factor (TF) families identified in the co-expression network with correlation coefficient cutoff of 0.8. Figure S1: Heat map of Pearson correlation coefficients among 36 samples. Figure S2: Principal Component Analysis of 36 samples. Figure S3: Correlation between RNA-seq (FPKM) and qRT-PCR expression values for six flavonoid-biosynthesis-related genes in *D*. *huoshanense*. Figure S4: Full co-expression network (|r| ≥ 0.8).
