# Supplementary material for: Time-Series Transcriptome Reveals Differential Regulation of Flavonoid Biosynthesis in Dendrobium huoshanense Under Musical–Acoustic Stimuli: Insights for Green Horticultural Production
Source: Genes (Basel). 2026 Sep 24;17(10):1178. doi: 10.3390/genes17101178 (PMC13704637; doi:10.3390/genes17101178)
Supplement: Supplementary file 1 [file genes-17-01178-s001.zip › Figure S3.pdf]

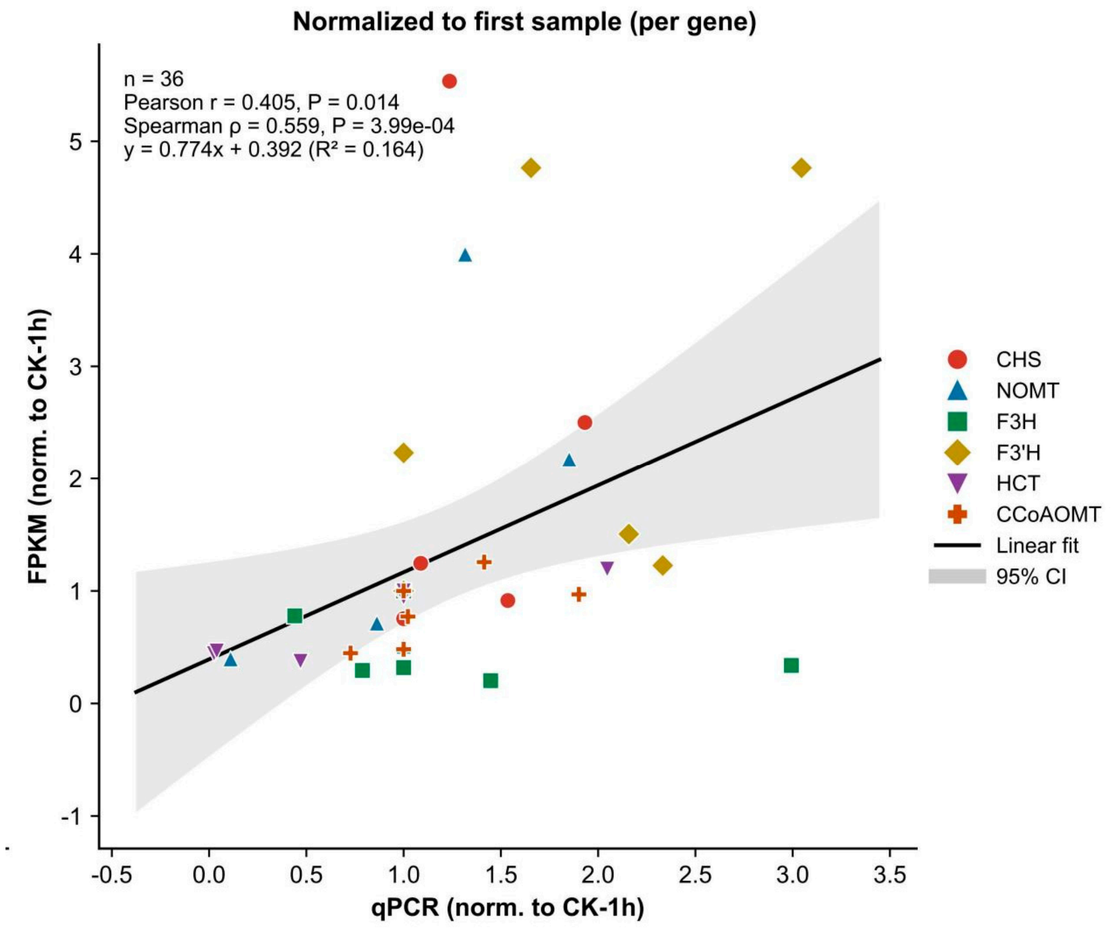

Figure S3 Correlation between RNA-seq (FPKM) and qRT-PCR expression values for six flavonoid-biosynthesis-related genes in *Dendrobium huoshanense*. For each gene, all expression values were normalized to the time-matched control at 1 h (CK-1h), yielding a total of n = 36 paired observations across four sampling time points (1, 6, 12, and 24 h) and three biological replicates. Different colored symbols represent individual genes: red circles, *CHS*; blue triangles, *NOMT*; green squares, *F3H*; yellow diamonds, *F3'H*; purple inverted triangles, *HCT*; orange crosses, *CCoAOMT*. The solid black line indicates the linear regression fit ($y = 0.774x + 0.392$, $R^2 = 0.164$), and the shaded grey band denotes the 95% confidence interval (CI) of the regression. Both Pearson's product-moment correlation (r = 0.405, P = 0.014) and Spearman's rank-order correlation (ρ = 0.559, $P = 3.99 \times 10^{-4}$) reached statistical significance, supporting the overall concordance of expression trends between RNA-seq and qRT-PCR platforms.
