# Supplementary material for: Time-Series Transcriptome Reveals Differential Regulation of Flavonoid Biosynthesis in Dendrobium huoshanense Under Musical–Acoustic Stimuli: Insights for Green Horticultural Production
Source: Genes (Basel). 2026 Sep 24;17(10):1178. doi: 10.3390/genes17101178 (PMC13704637; doi:10.3390/genes17101178)
Supplement: Supplementary file 1 [file genes-17-01178-s001.zip › Figure S4.pdf]

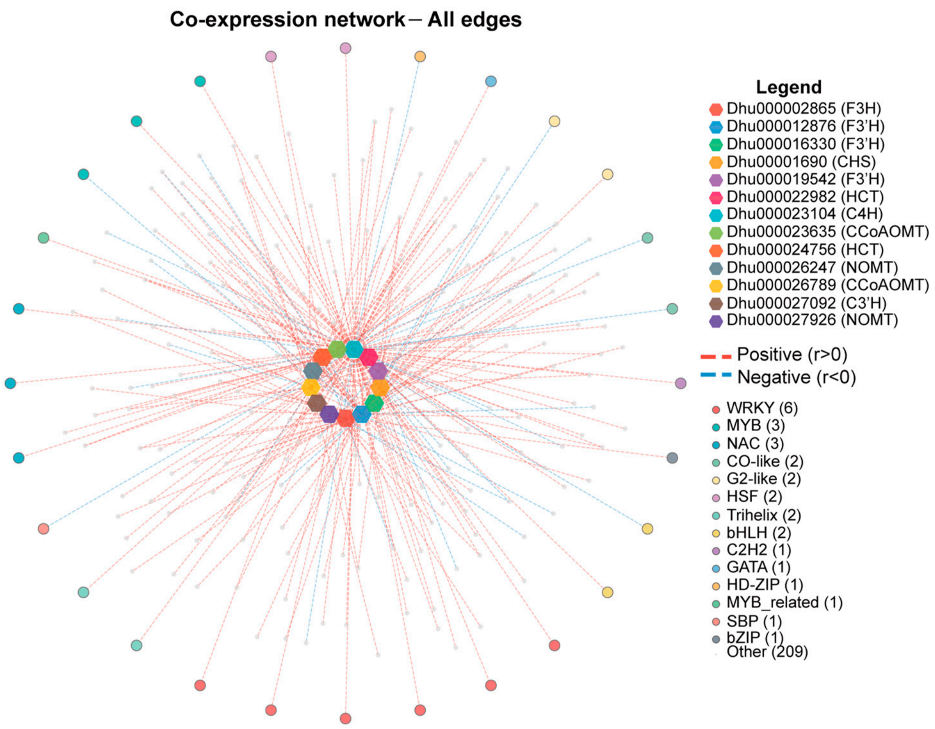

Figure S4 Full co-expression network (|r| ≥ 0.8). Central nodes: 13 flavonoid-biosynthetic hub genes; outer nodes: transcription-factor and non-TF genes. Red/blue dashed edges stand for positive/negative correlations.
